# Identification of high risk areas for avian influenza outbreaks in California using disease distribution models

**DOI:** 10.1371/journal.pone.0190824

**Published:** 2018-01-31

**Authors:** Jaber Belkhiria, Robert J. Hijmans, Walter Boyce, Beate M. Crossley, Beatriz Martínez-López

**Affiliations:** 1 Center for Animal Disease Modeling and Surveillance, Department of Medicine & Epidemiology, School of Veterinary Medicine, University of California, Davis, California, United States of America; 2 Department of Environmental Science & Policy, University of California, Davis, California, United States of America; 3 Department of Pathology, Microbiology & Immunology, School of Veterinary Medicine, University of California, Davis, California, United States of America; 4 California Animal Health and Food Safety Lab, School of Veterinary Medicine, University of California, Davis, California, United States of America; University of Reunion Island, RÉUNION

## Abstract

The coexistence of different types of poultry operations such as free range and backyard flocks, large commercial indoor farms and live bird markets, as well as the presence of many areas where wild and domestic birds co-exist, make California susceptible to avian influenza outbreaks. The 2014–2015 highly pathogenic Avian Influenza (HPAI) outbreaks affecting California and other states in the United States have underscored the need for solutions to protect the US poultry industry against this devastating disease. We applied disease distribution models to predict where Avian influenza is likely to occur and the risk for HPAI outbreaks is highest. We used observations on the presence of Low Pathogenic Avian influenza virus (LPAI) in waterfowl or water samples at 355 locations throughout the state and environmental variables relevant to the disease epidemiology. We used two algorithms, Random Forest and MaxEnt, and two data-sets Presence-Background and Presence-Absence data. The models performed well (AUCc > 0.7 for testing data), particularly those using Presence-Background data (AUCc > 0.85). Spatial predictions were similar between algorithms, but there were large differences between the predictions with Presence-Absence and Presence-Background data. Overall, predictors that contributed most to the models included land cover, distance to coast, and broiler farm density. Models successfully identified several counties as high-to-intermediate risk out of the 8 counties with observed outbreaks during the 2014–2015 HPAI epizootics. This study provides further insights into the spatial epidemiology of AI in California, and the high spatial resolution maps may be useful to guide risk-based surveillance and outreach efforts.

## Introduction

The 2014–2015 Highly pathogenic Avian Influenza (HPAI) epizootics in the United States of America (USA) were one of the largest HPAI outbreaks affecting poultry in the country’s history and one of the most economically and socially devastating [1]. The full chronology of the epizootics remains unknown, but it appears that that they triggered by the introduction into North America of an intercontinental clade (icA) 2.3.4.4 H5N8 by migrating waterfowl [2]. California’s poultry farms were among the first to be affected [3]. The state is a large poultry producer and a leader in the free-ranging chicken industry in the USA [4]. California’s Central Valley also attracts a very large number of overwintering waterfowl [5]. Three pathways for initial occurrence of HPAI outbreaks in poultry farms California have been suggested. The first is via migratory waterfowl that harbor a variety of Avian Influenza Viruses (AIV) such as the (icA) clade 2.3.4.4 H5N8 which could be directly introduced into poultry farms, as what likely happened during the 2014–2015 outbreaks. Clade 2.3.4.4 H5N8 (icA) was detected in Stanislaus County in 14-week-old commercial broad-breasted white turkeys in January 2015 followed by a second outbreak in February 2015 in 12-week-old chickens in Kings County [3]. A second pathway is the appearance of new reassortant HPAI viruses (HPAIv). Reassortant HPAIv are the result of the intermixing of the hemaglutinin gene from an HPAIv and/or a neuraminidase gene of a North American (NA) Low Pathogenic Avian Influenza Virus (LPAIv) in the same host [6]. The reassortant HPAIv H5N2 (NA) that spread throughout the midwestern USA is a good example [7]. HPAIv H5N2 (NA) was not detected in Californian poultry farms [8]; however, its introduction could have led to massive outbreaks as occurred in the state of Iowa with the reassortant HPAIv H5N2 (NA) [1]. The third pathway suggested is that LPAIv introduced into a poultry farm could have a series of mutations that lead to novel HPAIv’s generating new outbreaks [9,10].

Immediately after the detection of the first HPAI outbreak (2014), the United States Department of Agriculture (USDA) and the California Department of Food and Agriculture Department (CDFA) responded by enhancing AI surveillance along bird migration routes (the Pacific Flyway) [11]. The early detection of AI, targeting high risk areas for the three pathways mentioned above, are key to prevent HPAI epizootics. Identifying high risk areas for HPAI could assist in sampling and planning of education and outreach programs and help to create a sensitive and cost-effective surveillance system.

A recent study used a disease distribution model (DDM) to determine suitable areas for AI presence in the USA using Presence-Background data [12]. Disease distribution models’ main outcomes are maps that identify the environmental similarity of a location to areas where the disease has been observed. Sites "similar" to those where the disease (or pathogen) was observed are generally expected to have a higher probability of AI occurrence than other locations [13–17]. We use the term ‘suitability’ to express the degree of environmental similarity of a site to sites where the disease was observed. This study expands and refines this work [12] for California. Our aim is to generate more precise maps of suitable areas for AIV in California taking into account the three HPAI pathways described. LPAI data from both wild birds and from water samples were used in models with multiple disease-specific environmental predictors. To address two major sources of uncertainty in this type of modeling, we evaluated four modeling approaches resulting from the combination of two algorithms -MaxEnt and Random Forest- and two data sets -presence-background (P-B) and presence-absence (P-A). Results emerging from this study could be helpful to prevent future HPAI outbreaks, as we highlight not only specific factors playing a role in AIV dynamic in California but also areas suitable for AIV presence and thus at higher risk for initial AI outbreaks in poultry farms as well as “hot-spots” for waterfowl.

## Methods

### Data source

LPAI presence-absence (LPAI P-A) data consisted of water and wild bird samples. Wild bird LPAI P-A samples for April 2007 to September 2016 were extracted from the Influenza Research Database (FluDB) [18]. FluDB data contain information on bird AI samples, including the sampling locations’ coordinates, species, AI-testing results, and viral subtypes. Samples without location coordinates (2%) or AI test results were disregarded. Duplicate samples in a same exact location were aggregated. A location was classified as LPAI positive if at least one positive sample was collected there. Water P-A samples were collected in 2014 from artificial water ponds across California in a previous study [19,20]. Water P-A samples contained both the geographic locations of every sample and laboratory results confirming the presence or absence of AIV, based on an RT-PCR targeting the matrix gene sequence of the AIV [19].

**Fig 1:**
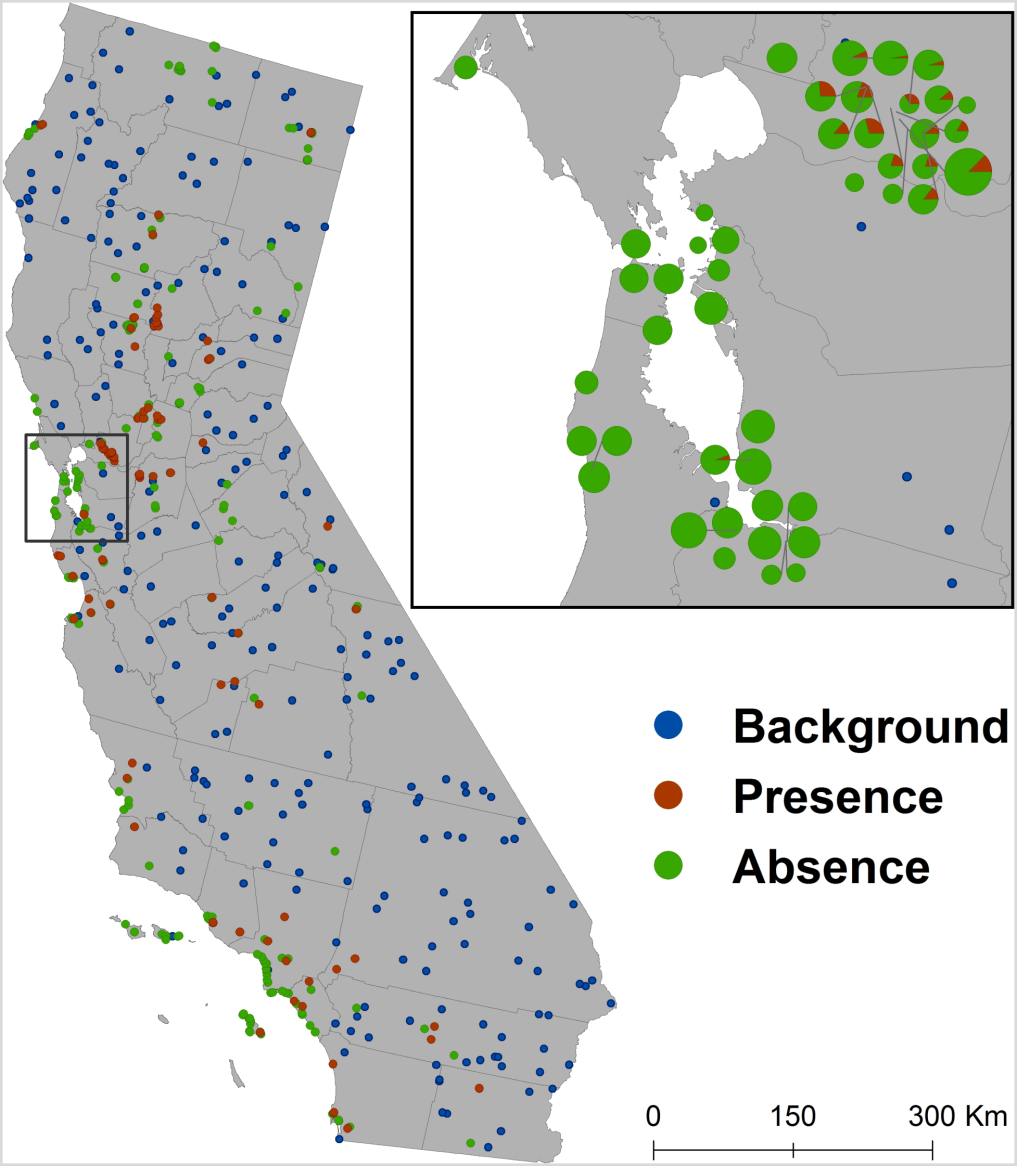
Spatial distribution of presence and absence and background samples. The zoomed area presents an example of the raw samples’ distribution before data cleaning.

Raw data extracted from FluDB consisted of 19.217 samples. See the supplementary information for details (S1 Table, S1 and S2 Figs). The final dataset combining both waterfowl and water samples consisted of 351 unique sites for which we had presence or absence (Fig 1). Samples with identical locations were eliminated and geographic coordinates matching with at least one positive case, were considered as positive locations. The highlighted area in Fig 1 presents an example of the raw data distribution before the adjustment. Out of the 351 sites, 182 (52%) were water sample sites and 169 (48%) were wild bird sample sites. A total of 110 samples (31%) were positive out of which 45 samples (41%) were from water samples and 65 (59%) were from wild birds.

We created a set of randomly selected background (sometimes referred to as ‘pseudo-absence’) locations, excluding sites that had positive samples. A site was defined as a 500 × 500 m grid cell. To match the absence data, 241 background point locations were randomly drawn (Fig 1).

We selected a set of environmental predictors that have been previously described as important factors in understanding the presence/absence of AI [12,21–23]. These predictors were also selected to fit the three HPAI occurrence pathways described in California. Predictors were organized as raster data with a cell size of 500 × 500m and the Albers Equal-Area coordinate reference system centered on California (“California Albers”) was used.

Bioclimatic variables were extracted from the WorldClim database [24]. Landscape variables used included elevation, Normalized Difference Vegetation Index (NDVI), land cover, important farmland in California (IFC), and distances to both lakes and coastline. The average NDVI over the study period was derived from reflectance measured by the Moderate Resolution Imaging Spectroradiometer (MODIS) aboard NASA’s Terra satellite. The California land cover data were obtained from the CALFIRE database [25]. The different categories of land cover considered are provided in the S2 Table. Important farmland in California (IFC) is a classification of the different farmland categories by the California Department of Conservation under the Farmland Mapping and Monitoring Program (FMMP) [26]. The different categories of farmland considered are provided in the S3 Table.

Bird related predictors consisted of poultry farm density (by type of production) system and the Important Bird and Biodiversity Areas (IBA) as a proxy for the presence of migratory birds. Poultry farm densities included as separate predictors were broiler, turkey, pullet, duck, geese and backyard poultry. Methods used to create these rasters are explained elsewhere [12]. The IBA data [27] identifies 149 areas covering about 40,000 km^2^ in California that provide essential habitat for breeding, wintering and shelter for migratory birds. Distance to open water and coastline were computed using ArcGIS 10.3 [28], the inland water points from the US. Geological Survey [29] and the coastline data from the Natural Earth database [30].

### Analysis: Disease distribution models (DDM)

We used both the MaxEnt and RandomForest algorithms to model the AI data. The two algorithms were chosen because they are commonly used in species distribution modeling, as both have been found to have high prediction accuracy [31] [32]. MaxEnt [33], which uses a maximum entropy classifier was run using the “dismo” package in the R software [34]. Random Forest (RF) is an extension of Classification and Regression Trees (CART). Random Forest consists of a combination of many trees (CART models), where each tree is generated with a bootstrapped sample (i.e., a sample with replacement of the same size as the dataset) thus taking about 63% of the observations for each tree. Each split of the tree is determined using one variable selected from a random subset of the potential variables. The final prediction is the average prediction of all the trees [35,36]. Random Forest analyses were carried out using the “RandomForest” package in R [37]. MaxEnt and RF were consecutively trained with Presence-Absence and Presence-Background data to generate four different models: MaxEnt presence-absence (MPA), MaxEnt presence-background (MPB), RF presence-absence (RFPA) and RF presence-background (RFPB).

For each MaxEnt model, we determined the most important variables with both the Jackknife training gain and the percent contribution, both are measures of how much a variable contributed to the model.

To obtain stable results with RF, we populated a forest with 1001 trees (ntree = 1001) sampling 4 variables at random for each of the nodes in each tree. To select the most relevant variables to include in the final model we ranked the variables according to their importance. We used Mean Decrease in Accuracy (MDA) for variable importance measure available in RF since it’s considered a more reliable measure than the decrease in node impurity [38]. This measure corresponds to the difference between the misclassification rate for the original and the permuted out-of-bag samples (i.e., not included in the bootstrapped sample for a particular tree), averaged over all the trees and divided by the standard deviation of the differences.

We eliminated highly correlated predictor variables using Spearman’s rank correlation. Variables with a correlation of 0.6 or higher were considered highly correlated, and the variable that seemed least relevant for AI prediction was removed.

Non-correlated predictors were first organized in three groups (bioclimatic, farm densities and environmental factors). We ran initial selection models (MPA, MPB, RFPA and RFPB) for each group. The top three contributing variables in each group were then included in initial “full” models, respectfully, for MPA, MPB, RFPA and RFPB. Subsequently, variables that contributed most to a model were retained and used for “final” models. The four final models were evaluated and used for prediction. Model predictions were scaled from 0 to 1 by first subtracting the minimum value, and then dividing by the maximum value.

Suitability scores from the four models were projected over the study space in the shape of four risk maps. Since the Presence-Absence dataset was collected exclusively from waterfowl habitats, risk maps from Presence-Absence models could be seen as contrasting a sub-domain (wild bird habitat) from the entire study areas instead of presenting the true areas at risk for AI outbreaks. To account for this, we created a corrected version of the Presence-Absence risk maps (“MPAc” and “RFPAc”). MPAc and RFPAc risk maps represent the product of the probabilities of the original MPA and RFPA with the probabilities from “sampling site” models. The sampling site models with MaxEnt and RF contrast all sampling sites with background randomly selected from the entire study area.

### Model evaluation

We assessed the model fit (with testing data) and compared the predicted disease suitability distributions to recent outbreaks [39]. We used 5-fold cross-validation to compute the area under the receiver-operating characteristic curve (AUC) and the corrected AUC (AUCc). AUC is a normalized measure of model fit that can be derived from Wilcoxon’s rank-correlation statistic, with values that can range from 0 to 1. A model performs well when the AUC is close to 1. Usually, AUC values of >0.9 indicate high accuracy, values between 0.7 to 0.9 indicate good accuracy, and values between 0.6 to 0.7 indicate low accuracy. A value of 0.5 would suggest that a prediction is no better than a random guess. Because of spatial autocorrelation, AUC is affected by the geographic distance between model training and testing sites (spatial sorting bias). We corrected for this by using the calibrated AUC (AUCc) instead of the regular AUC [40]. AUCc were also used to generate weighted mean risk maps for P-A models (MPA and RFPA) and for the P-B models (MPB and RFPB). Weights for each model *i* (*W*_*i*_) were computed using AUCc of each model using Equation (1):

$$W_{i}={\left(AUCc_{i}-0.5\right)}^{2}$$

We used the USDA official notification data on 2014–2015 HPAI outbreaks to evaluate the generated AI risk maps. To compare the spatial predictions of the different models, we computed Spearman’s correlation coefficients between the predictions for the 500 × 500 m raster cells in California (n = 3,301,320). Because HPAI outbreak locations are only available at the county level, we estimated the mean AI risk per county for each DDM. Counties were then classified by terciles as “Low Risk” “Moderate Risk” or “High Risk”. A HPAI outbreak was considered as "correctly detected" by either MaxEnt or RF if it was located in a “High Risk” county.

## Results

**Fig 2:**
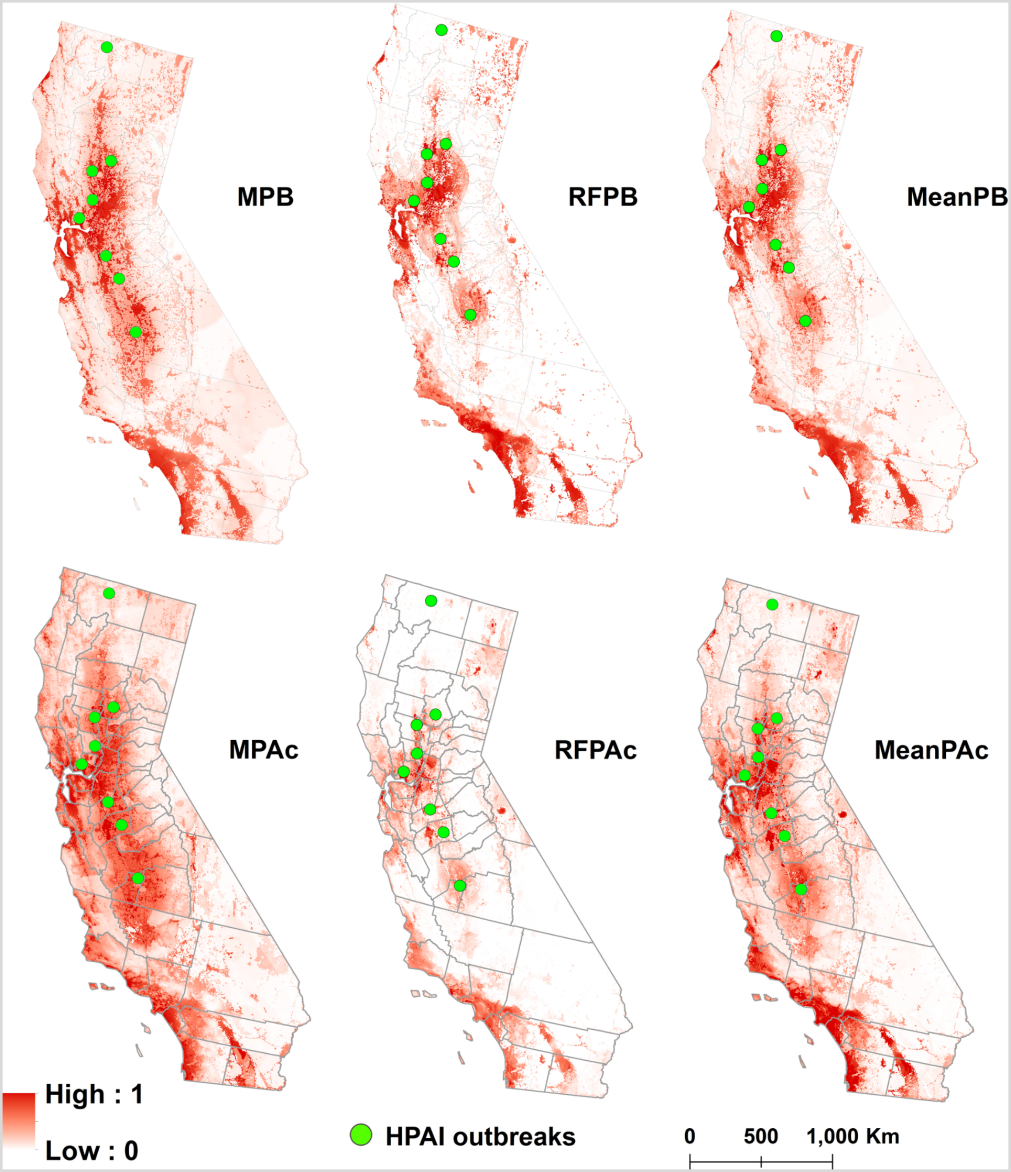
Risk maps generated from the four diseases distribution models and their means: Presence-background MaxEnt (MPB), presence-background Random Forest (RFPB), weighted mean of both presence-background models based on AUCc (MeanPB), corrected presence-absence MaxEnt (MPAc), corrected presence-absence Random Forest (RFPAc), weighted mean of both presence-absence models based on AUCc (MeanPAc). The green dots represent the centroids of the 2014–2015 HPAI outbreaks. The color gradient of each pixel represents the AI presence probability from clear red shading (low presence probability) to bright red shading (high presence probability). High resolution versions of the maps are available in Disease BioPortal (http://bioportal.ucdavis.edu).

Overall, all models performed well (AUCc > 0.7 for testing data), particularly for models trained with P-B data (AUCc > 0.85) (Table 1). The original spatial predictions generated from MPA, RFPA and the “sampling site” models spatial predictions are presented in the S3 Fig. Spatial predictions generated by MaxEnt and RF were very similar for the P-B data (rho = 0.81) but less so for the P-A data (rho = 0.65). There were large similarities between the P-A and P-B model predictions irrespective of the used algorithm (Fig 2, Table 2). While P-B models predicted more areas with extreme high or low values, P-A models, particularly MPA, predicted a diffused medium/low values for large areas. Areas with high values for P-B also had high values for P-A.

**Table 1:** AUC and AUCc values for the four DDMs: Maxent Presence Bakground (MPB), Maxent Presence-Absence (MPA), Random Forest Presence Background (RFPB) and Random Forest Presence-Absence (RFPA).

	MPB	MPA	RFPB	RFPA
AUC	0.95	0.86	0.95	0.74
AUCc	0.93	0.85	0.78	0.68

**Table 2:** Spearman’s correlation coefficient between the spatial predictions of the for the four DDMs, Maxent Presence Background (MPB), Corrected Maxent Presence-Absence (MPAc), Random Forest Presence Background (RFPB), Corrected Random Forest Presence-Absence (RFPAc) and, the model averages for Presence-Absence (WPAc) and Presence-Background (WPB) of Avian Influence occurrence in California, USA. Predictions were made for 500 × 500 m grid cells in California (n = 3,301,320).

	MPB	MPAc	RFPB	RFPAc	WPB
MPAc	0.85				
RFPB	0.82	0.70			
RFPAc	0.70	0.65	0.81		
WPB	0.93	0.79	0.97	0.80	
WPAc	0.83	0.85	0.84	0.95	0.87

The most important contributing predictors for the MPB model were elevation (52% contribution), land cover (30%), broiler farm density (14%) and distance to the coast (4.2%) (Table 3). The AUCc of this MaxEnt model was 0.93. High risk areas (> 0.6) according to the MPB model are mostly around the San Francisco Bay Area, the Central Valley and Southern California (coastal and the Imperial and Coachella Valleys) (Fig 2). The MPB model classified 2 out of the 8 counties where HPAI outbreaks were reported as high risk, 5 as medium risk and 1 as low risk (Fig 2).

**Table 3:** Variable importance for the final models of the four DDMs: Maxent Presence Background (MPB), Maxent Presence-Absence (MPA), Random Forest Presence Background (RFPB) and Random Forest Presence-Absence (RFPA) of Avian Influence occurrence in California, USA. Variable importance was determined with percent contribution for Maxent and mean decrease in accuracy for Random Forest.

Importance	MPB	MPA	RFPB	RFPA
1	Elevation (52%)	Distance to the coast (42.5%)	Land cover (98.2)	Broiler farm density (41.3)
2	Land cover (29.6%)	Backyard farms density (24.7%)	Minimum temperature of coldest month (57.5)	IBA (29)
3	Broiler farm density (14.2%)	Land cover (18.2%)	Precipitation Seasonality (32.0)	Distance to the coast (26.7)
4	Distance to the coast (42%)	Precipitation of the driest quarter (14.7%)	Broiler farm density (30.8)	Min temperature of coldest month (24.2)
5	-	-	-	Land cover (20)

The most important variables for the RFPB model were land cover (MDA = 98.2), minimum temperature of coldest month (MDA = 57.5), precipitation seasonality (MDA = 32.0) and broiler density (MDA = 30.8) (Table 3). The out-of-bag estimate of error rate was 11.9%. The AUC of the selected model was of 0.95 and the AUCc was 0.78. The risk map again pointed at the Bay Area, the Central Valley, parts of southern California, as well as the Central Coast as highly suitable for AIV whereas the Eastern Sierra Valley region are at low risk for the virus (Fig 2). The prediction map for the RFPB model identified 2 out of the 8 counties where HPAI outbreaks were reported, 5 in intermediate risk counties and 1 in low risk counties (Fig 2).

The most important contributing predictors for the MPA were distance to the coast (42.5% contribution), backyard farm density (24.7%), land cover (18.2%) and precipitation of driest quarter (14.7%) (Table 3). The AUC value of this MaxEnt model was 0.86 and the AUCc was 0.85. Both the San Francisco Bay Area and the Central Valley were at high risk for outbreaks. Few other locations in Northern California, the Coastal Region and Southern California were also designated as high risk (Fig 2). Overall, the MPA model identified 4 out of the 8 counties where HPAI outbreaks were reported in 2014–2015 epizootics, 2 in intermediate risk counties and 2 in low risk counties (Fig 2)

The five most important predictors in the RFPA model were broiler density (MDA = 41.3), IBA (MDA = 29), distance to the coast (MDA = 26.7), minimum temperature of coldest month (MDA = 24.2) and land cover (MDA = 20) (Table 3). The OOB estimate of error rate was 30.7%. The AUC of the selected model was of 0.74 and the AUCc was 0.68. High risk areas were located mostly in the San Francisco Bay Area, the Central Valley and the Coastal Region. A few other high risk areas were observed in parts of Northern and Southern California (Fig 2). The RFPA model identified 4 out of the 8 HPAI outbreaks in high suitable counties, 3 in intermediate suitable counties and 1 in low suitable counties (Fig 2).

Variable response curves, correlation matrices and Jackknifes graphs for the selected variables in each model are presented in the S4, S5 and S6 Figs.

## Discussion

The use of 2 different algorithms (MaxEnt and Random Forest), both parameterized with P-B and P-A data allowed to compare these different approaches, get a better understanding of AI dynamics in California and generates state-wide risk maps for AI following the three HPAI introduction pathways into poultry farms. All models (MPB, RFPB, MPA and RFPA) indicate an overall high accuracy predicting AIV occurrence in California (AUCc > 0.7). The resulting maps showed agreement in classifying specific areas as very suitable for AI particularly in coastal and the inland valleys of southern California (particularly in Orange, Los Angeles, San Diego, Imperial and Ventura counties), in and near the San Francisco Bay area and delta (Alameda, Contra Costa, Napa, Santa Cruz, San Francisco, San Mateo, Solano, Sonoma and Sutter counties), and adjacent areas in the Central Valley (Sacramento and San Joaquin counties). We consider these areas as important targets for surveillance and the application of preventive measures against HPAI.

Several predictors were present in many of the final models, highlighting their importance for predicting AI risk in California following the three introduction pathway. More specifically, land cover, distance to the coast, and broiler farm density which largely contributed to all DDMs (Table 3). Land cover, particularly the presence of wetlands and broad-leaf forests, were the highest contributors to the models (S5 Fig). This confirms similar findings in a previous study in the Pacific Flyway [12]. Wetlands are very important to consider when looking at AIV dynamics. Migratory birds often follow waterways and/or the coast, but for waterfowl the presence of wetlands is also important. Wetlands provide the main habitat for (migrating) waterfowl which could be carrying one or multiple AIV. They also offer the perfect conditions for a longer viral presence in the enviornment [41]. Broiler farm density has been looked at as potential predictor for HPAI occurrence, particularly in Asia [42], where farming conditions are different from those in California. In Europe and Canada, where farming systems are more similar to California, broiler farms are known to be at a lower risk than other types of poultry farming [43]. The fact that broiler farms density plays an important role in the models could be explained by two different scenarios: (i) migratory waterfowl potentially carrying AIV (LP or HP) are attracted to areas dense with broiler farms possibly due to the presence of feed and/or domestic fowls raised in open spaces; AIV could then be introduced into the farms via direct/indirect contact and lead to AI outbreaks; (ii) oversampling in areas that are highly dense for poultry farms (i.e. sampling bias) could lead to more positive cases causing models to select broiler farm density as an important predictor even though it doesn’t not affect AI transmission. While AI outbreaks in broiler farms as a result of contact between domestic birds and waterfowl have been reported in North America and other areas [44,45], this scenario is less likely to occur in California. The state is among the top broiler producers in the country [46] and local authorities and producers are constantly reviewing biosecurity levels, minimizing the contacts between waterfowl and domestic birds [47]. However, broiler farms with low biosecurity or with open production systems may be at particular risk if risk mitigation strategies are not implemented.

Rainfall and minimum temperature also contributed in three of the four models (Table 3). This is consistent with similar studies that found that annual rainfall and temperatures are important to identify areas at high-risk areas for AIV outbreaks [48,49].

The algorithms’ spatial predictions largely agreed on the distribution of AIV risk in California with P-B data, less with P-A. Maxent and RF predictions both showed that the upper east part of California is not highly suitable for LPAI presence (Modoc, Lassen, Shasta, Plumas and Siskiyou county), although in the 2014–2015 HPAI epizootics cases were confirmed in wild birds in Siskiyou county. Model failure to identify Siskiyou county could be due to multiple reasons—for example, our models are calibrated to detect primary outbreak occurrence and thus could not detect secondary outbreaks or lateral spread. The main disagreement between MaxEnt and RF predictions is on the status of southeastern California, specifically San Bernardino, Riverside, Inyo and Imperial counties; the MaxEnt predictions for these areas were higher than the RF predictions.

When compared, models trained with P-B and P-A each showed each several strengths and weaknesses. P-B models had better AUCc (> 0.85) but models trained with P-A records were better at correctly labeling counties where the 2014–2015 HPAI outbreaks took place as “high risk” (4 out of 8 outbreaks: Kings, Merced, Stanislaus, Solano). P-A models predicted a much larger area to be at “high risk” (particularly with MaxEnt). P-B models, however, easily detected unsuitable areas such as the Sierra Nevada and Eastern California with little sampling effort.

It is common practice in ecological and epidemiological studies to use background rather than absence locations as these latter are rarely available [50], [51]. When using background points, if the sample selection bias is not accounted for, the distribution model might reflect more the sampling effort rather than the true presence distribution [52]. For this reason, P-A distribution models are believed to be preferable as they are less affected by sample selection bias. P-B models, however, could be more effective if sampling was directed towards areas where AI outbreaks are occurring. The P-A models produced higher risk scores in areas with very few sampling locations. Correcting the spatial predictions from the P-A models helped remove sampling bias (S3 Fig). Sampling rasters also confirmed the disparities in terms sampling efforts in California. However, it’s necessary to point that the generated P-A maps might be incomplete since the spatial predictions are only based on characteristics from sampled areas. Further sampling efforts particularly in new areas are necessary to get a full image of the real AI risk.

Samples from waterfowl and the environment are routinely collected for AIV surveillance making them a very useful dataset for suitability modeling. However, locations where environmental negative samples were collected might not reflect the true risk for AI outbreaks but rather echoes the site’s characteristics. For example, negative water samples may be associated with areas less used by waterfowl or with abiotic factors that reduce the ability of AIV to survive (pH, temperature and/or salinity for example). Positive locations were defined as areas where at least one positive bird was observed over the study period. Likewise, negative areas were locations where all sampled birds were negative. Waterfowl are mobile and one could think that positive birds could have been previously infected and stopped shedding virus by the time it was sampled. This could occur even if a negative location might offer all the environmental conditions supportive of AIV transmission, but positive birds were not sampled during the study period. We also assumed that all predictors and factors were stationary over time.

Accuracy of the models could be improved by using a truly state-wide sampling scheme and collecting samples from all migratory birds that could harbor AIV, and also including predictors reflecting farm biosecurity levels and exact locations of poultry farms. Exact locations of poultry farms, specifically backyard flocks could help improve predictions of future models. It would also be of interest to take seasonality and temporal variations more explicitly into consideration. For example, migratory birds are known to be important when modeling AI. Here we used IBA as a proxy for migratory bird density, but including densities of migratory wild birds by migratory seasons could give insights of locations and time periods that poses higher risk for AI transmission. An alternative approach could be used in the future to model the fraction of positive cases instead of eliminating samples with identical locations. It could also be of interest to analyze whether there are many false negatives due to small sample sizes at some locations using the probability of detection approaches as described in previous studies [53].

## Conclusion

During the 2014–2015 HPAI epizootics in the US, only two cases were detected in Californian commercial poultry farms; however, California environmental conditions are favorable for AIV presence, and thus future outbreaks (in poultry and waterfowl) are likely to occur. AI risk maps generated could help decision makers and local stakeholders in preventing HPAI outbreaks as they highlight areas at risk for initial viral introduction into poultry farms as well as hot spots for waterfowl virus shedding. Risk maps are valuable for supporting the design of risk-based surveillance, and also targeting education and outreach campaigns for producers and poultry veterinarians, with an ultimate goal is to help prevent and rapidly control future AI outbreaks to protect the California poultry industry.

## Supporting information

S1 Table Most important bird species sampled in the FluDB raw dataset. (DOCX)

S2 Table Land cover classes and their pixel values in the raster used in the models. (DOCX)

S3 Table Important farmland map classes and their pixel values in the raster used in the models. (DOCX)

S1 Fig Temporal sampling distribution of the FluDB raw dataset by month over the entire study period and by year. The blue color indicates the number of samples that tested negative and the red color represents the number of samples that tested positive.
(TIF)

S2 Fig Spatio-temporal sampling distribution per unique location of the FluDB raw dataset. The X axis indicates the year of sampling. The Y axis represents the coordinates of every single location sampled. The color gradient represents the percent of positive samples in that specific location per year.
(TIF)

S3 Fig Spatial predictions from the original presence-absence MaxEnt (MPA), presence-absence Random Forest (RFPA), their respective sampling probabilities (M sampling, RF sampling) and the corrected Spatial predictions for MaxEnt (MPAc) and Random Forest (RFPAc). The color gradient of each pixel represents the presence probability from clear red shading (low presence probability) to bright red shading (high presence probability).
(TIF)

S4 Fig Spearman Correlation plots for the four models (MPA, RFPA, MPB, RFPB). (TIF)

S5 Fig Response curves for each of the four models (MPA, RFPA, MPB, RFPB). (TIF)

S6 Fig Jackknife of regularized training gain of the variables included in the two MaxEnt models (MPA, MPB). The red bar represents the overall training gain with all the included variables. The blue bar represents the training gain when using each variable in isolation. The clear blue bar is the training gain when the variable is excluded from the model.
(TIF)
