# Supplementary material for: Identification of high risk areas for avian influenza outbreaks in California using disease distribution models
Source: PLoS One. 2018 Jan 31;13(1):e0190824. doi: 10.1371/journal.pone.0190824 (PMC5791985; doi:10.1371/journal.pone.0190824)
Supplement: S1 Table — (DOCX) [file pone.0190824.s001.docx]

| *Host.Species* | *Flu.Test.Status* | | ***Total*** |
| --- | --- | --- | --- |
|  | Negative | Positive |  |
| Aix sponsa | 296 | 2 | 298 |
| Anas acuta | 1492 | 39 | 1531 |
| Anas americana | 1251 | 54 | 1305 |
| Anas carolinensis | 655 | 14 | 669 |
| Anas clypeata | 1810 | 128 | 1938 |
| Anas crecca | 1214 | 34 | 1248 |
| Anas strepera | 1181 | 25 | 1206 |
| Aythya collaris | 253 | 9 | 262 |
| Larus californicus | 684 | 52 | 736 |
| Other | 9259 | 765 | 10024 |
| ***Total*** | 18095 | 1122 | 19217 |
|  | | | |
