# Supplementary material for: Identification of high risk areas for avian influenza outbreaks in California using disease distribution models
Source: PLoS One. 2018 Jan 31;13(1):e0190824. doi: 10.1371/journal.pone.0190824 (PMC5791985; doi:10.1371/journal.pone.0190824)
Supplement: S2 Table — (DOCX) [file pone.0190824.s002.docx]

| **Pixel Value** |  | **Land cover classes** |
| --- | --- | --- |
| 10 | Agriculture: Less than 10% natural vegetation and area is under cultivation. | |
| 20 | Barren/Other: Less than 10% vegetative cover | |
| 31 | Conifer Forest: Conifer Forest > 10% cover | |
| 32 | Conifer Woodland: Conifer Woodland > 10% cover | |
| 41 | Desert Shrub: Desert shrub vegetation > 3% and not conifer, hardwood, shrub | |
| 42 | Desert Woodland: Desert Woodland vegetation > 3% and not conifer, hardwood, shrub | |
| 51 | Hardwood Forest: Hardwood Forest > 10% cover and conifer < 25% | |
| 52 | Hardwood Woodland: Hardwood Woodland > 10% and conifer < 10% | |
| 60 | Herbaceous: Herbaceous > 10% cover and Tree/shrub < 10% cover | |
| 70 | Shrub: Shrub > 10% cover and tree < 10% cover | |
| 80 | Urban: Urban > 10% cover and native tree/shrub/grass < 10% cover | |
| 90 | Water | |
| 100 | Wetland: Wetland > 10% cover and not tree/shrub | |
