# Supplementary material for: Identification of high risk areas for avian influenza outbreaks in California using disease distribution models
Source: PLoS One. 2018 Jan 31;13(1):e0190824. doi: 10.1371/journal.pone.0190824 (PMC5791985; doi:10.1371/journal.pone.0190824)
Supplement: S3 Table — (DOCX) [file pone.0190824.s003.docx]

|  | |
| --- | --- |
| **Pixel Value** | **Important Farmland Map classes** |
| 1 | Urban and Built-Up Land |
| 2 | Grazing Land |
| 3 | Prime Farmland |
| 4 | Farmland of Statewide Importance |
| 5 | Unique Farmland |
| 6 | Water |
| 7 | Other Land |
| 8 | Area not mapped |
| 9 | Confined Animal Agriculture |
| 10 | Nonagricultural and Natural Vegetation |
| 11 | Rural Residential and Rural Commercial |
| 12 | Semi-Agricultural and Rural Commercial Land |
| 13 | Vacant or Disturbed Land |
| 14 | Farmland of Local Importance |
| 15 | Farmland of Local Potential |
