# Supplementary figures and images for: Identification of high risk areas for avian influenza outbreaks in California using disease distribution models
Source: PLoS One. 2018 Jan 31;13(1):e0190824. doi: 10.1371/journal.pone.0190824 (PMC5791985; doi:10.1371/journal.pone.0190824)

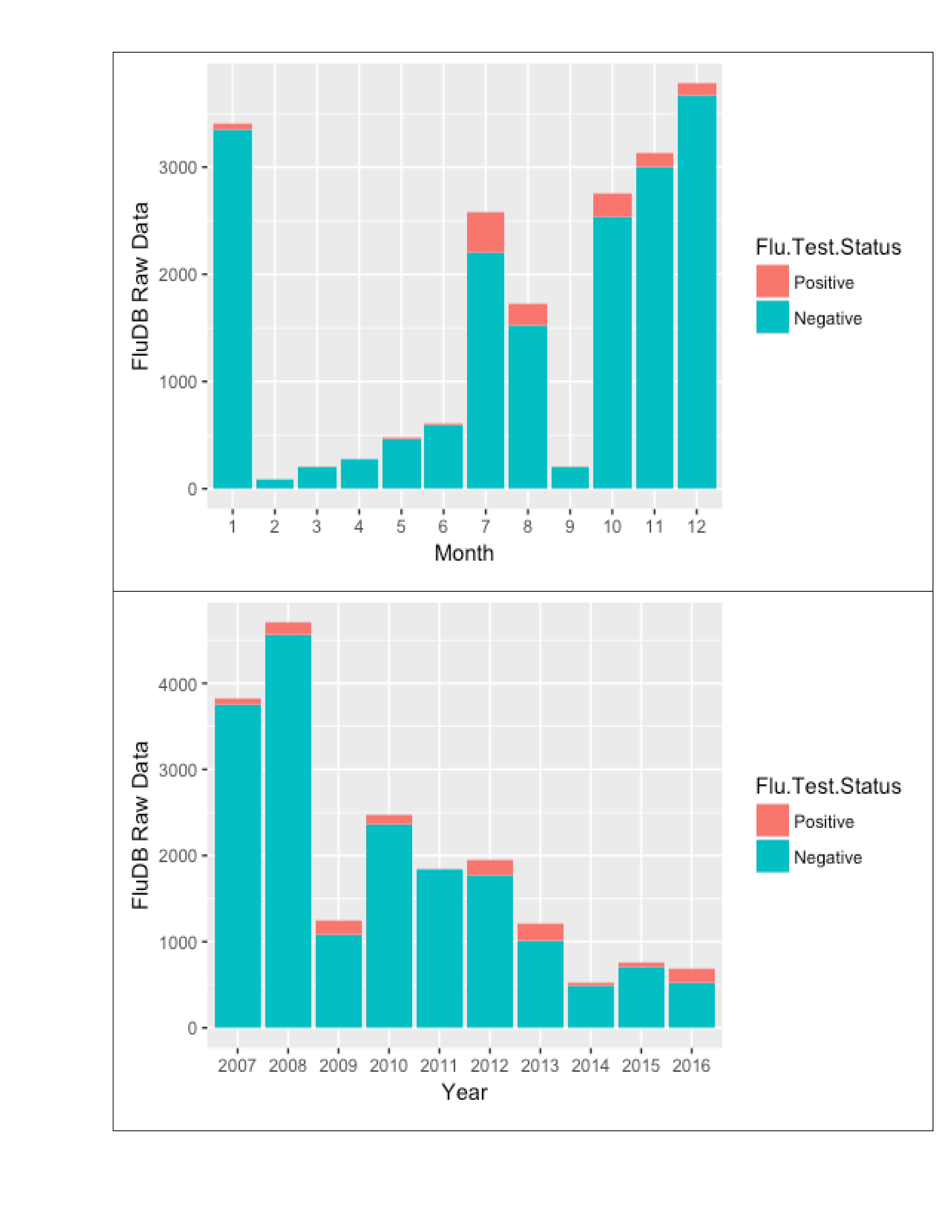

Supplement: S1 Fig — The blue color indicates the number of samples that tested negative and the red color represents the number of samples that tested positive. (TIF) [file pone.0190824.s004.tif]

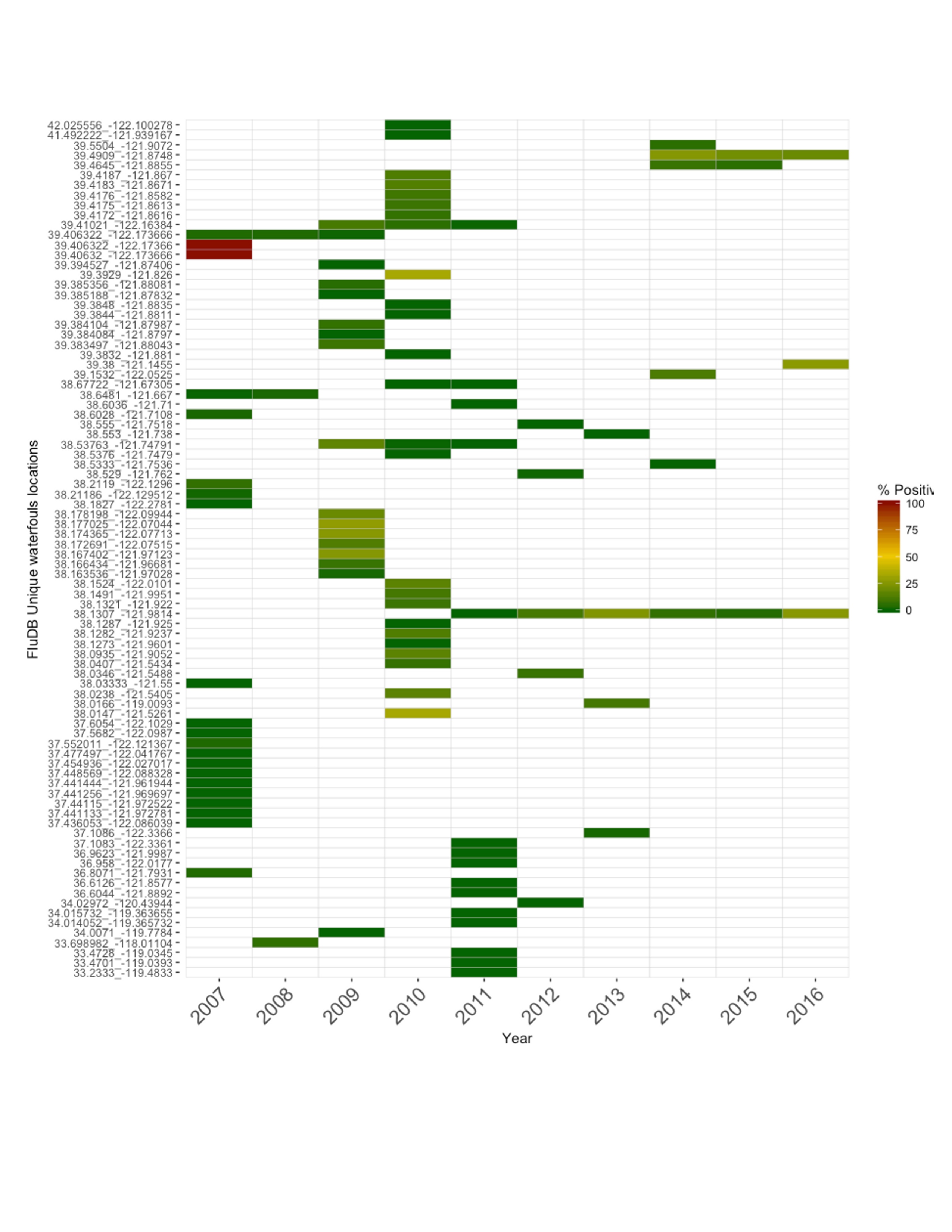

Supplement: S2 Fig — The X axis indicates the year of sampling. The Y axis represents the coordinates of every single location sampled. The color gradient represents the percent of positive samples in that specific location per year. (TIF) [file pone.0190824.s005.tif]

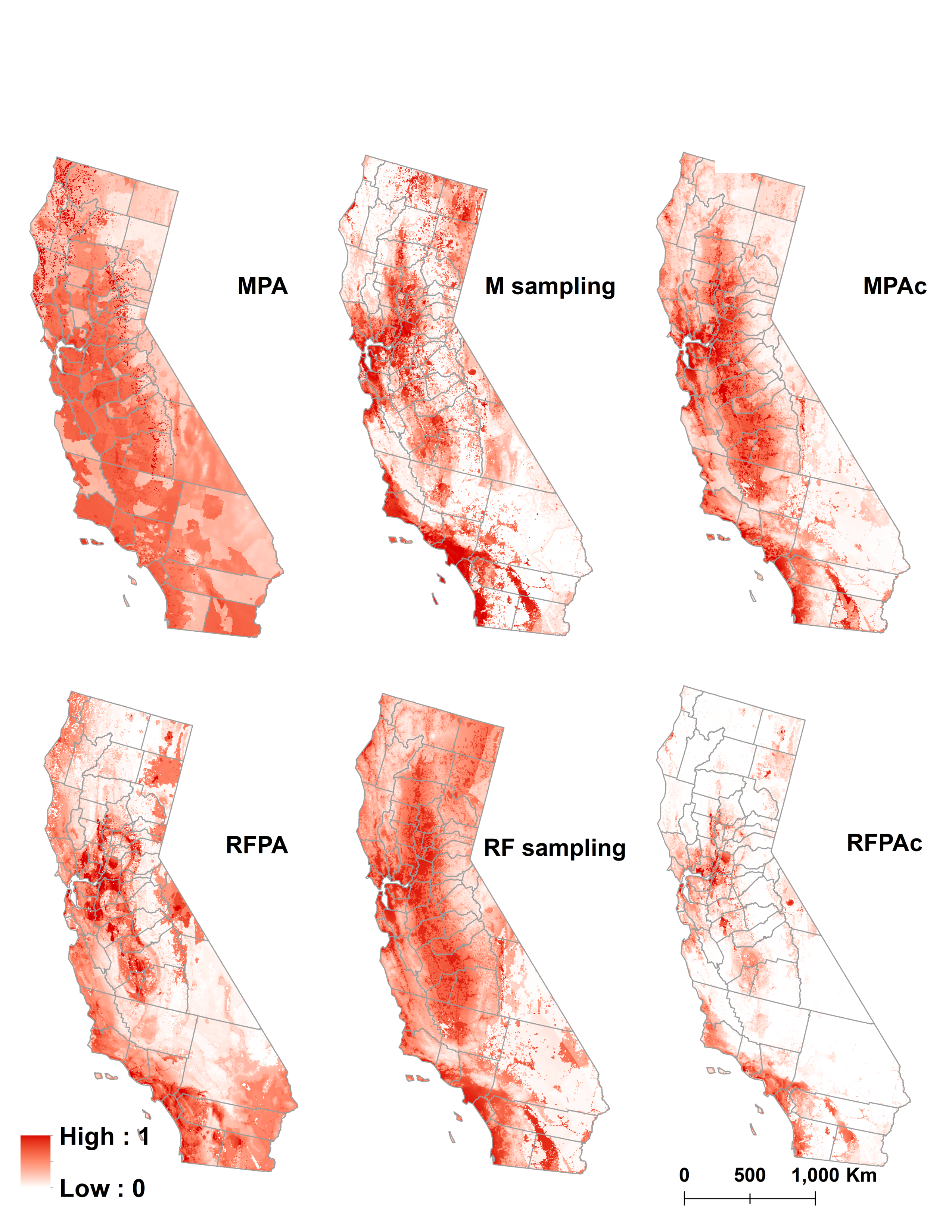

Supplement: S3 Fig — The color gradient of each pixel represents the presence probability from clear red shading (low presence probability) to bright red shading (high presence probability). (TIF) [file pone.0190824.s006.tif]

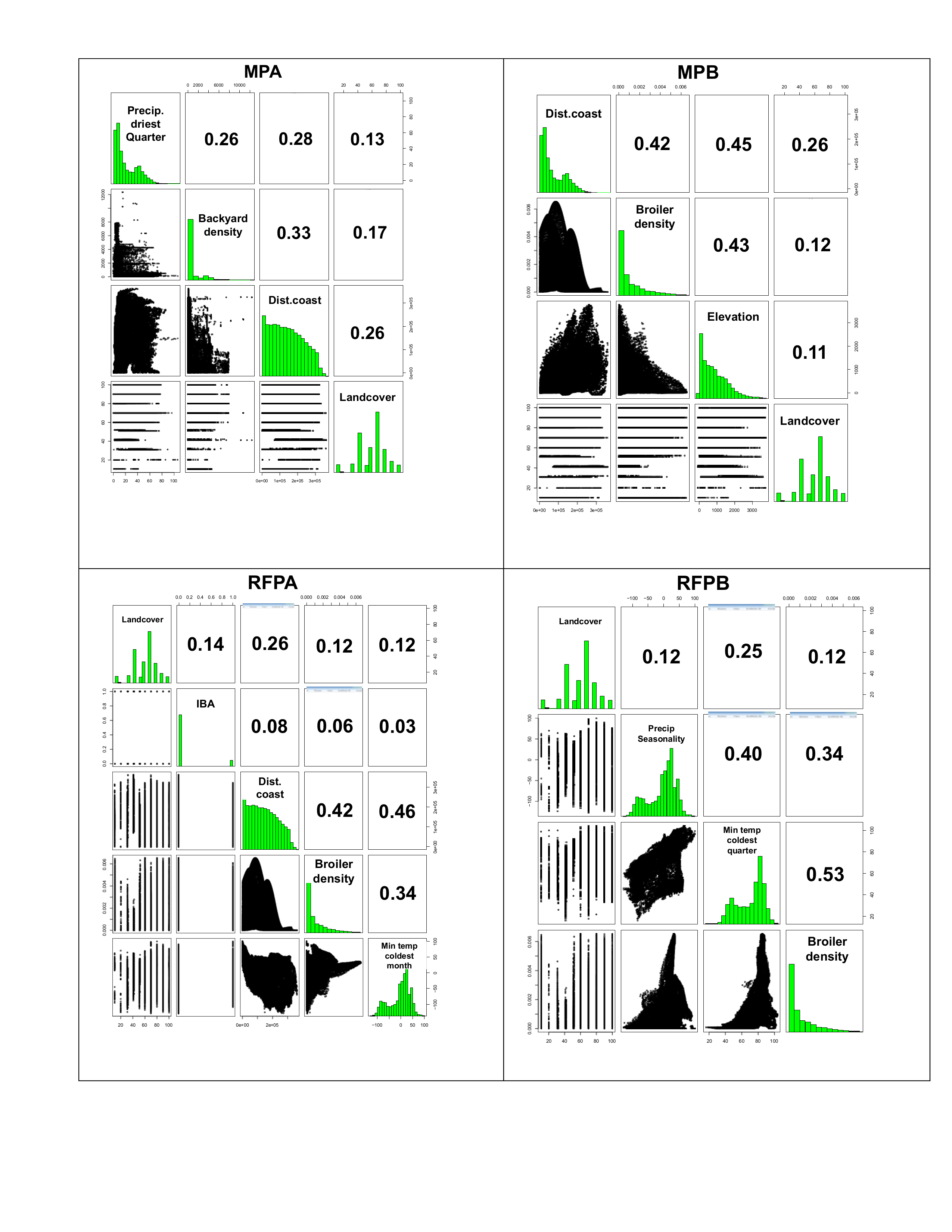

Supplement: S4 Fig — (TIF) [file pone.0190824.s007.tif]

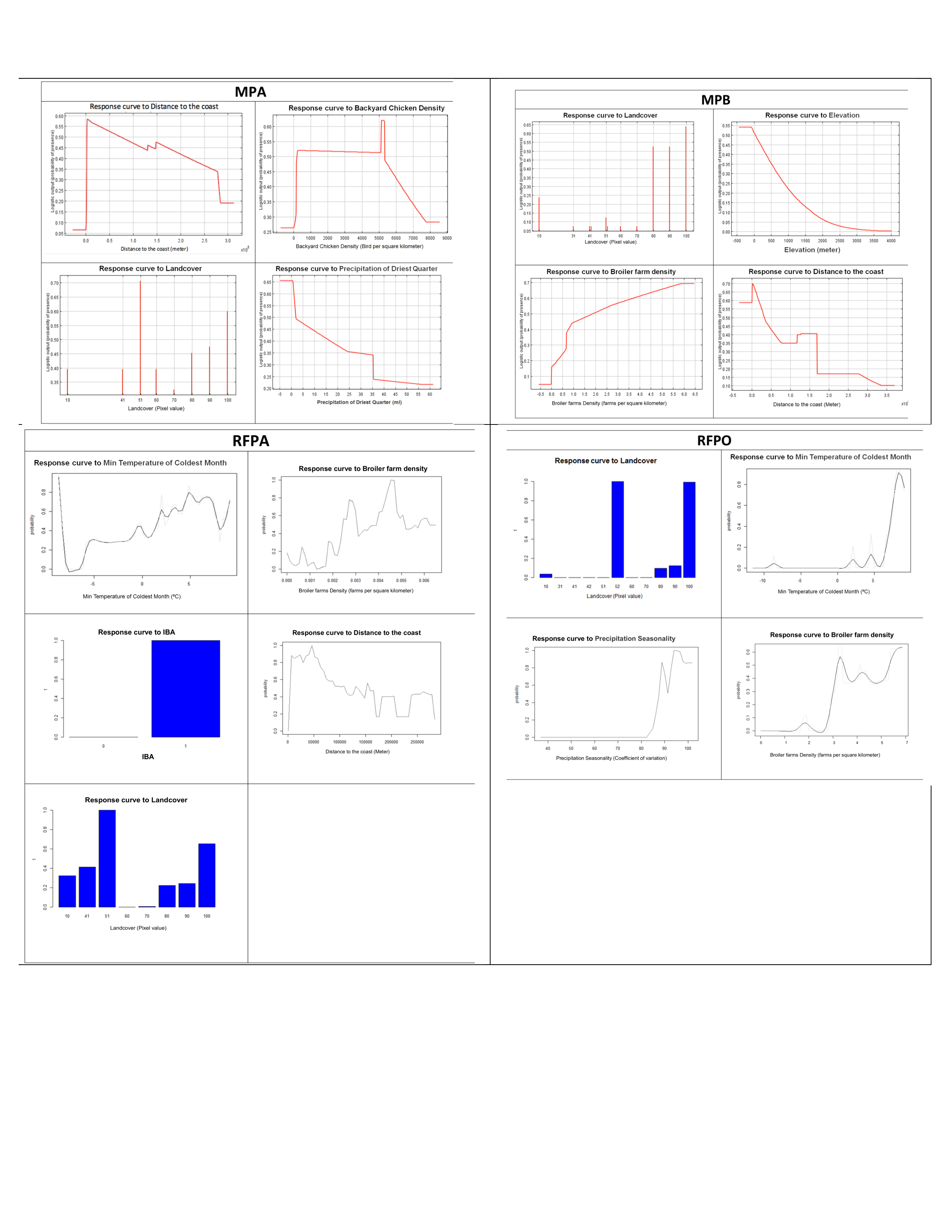

Supplement: S5 Fig — (TIF) [file pone.0190824.s008.tif]

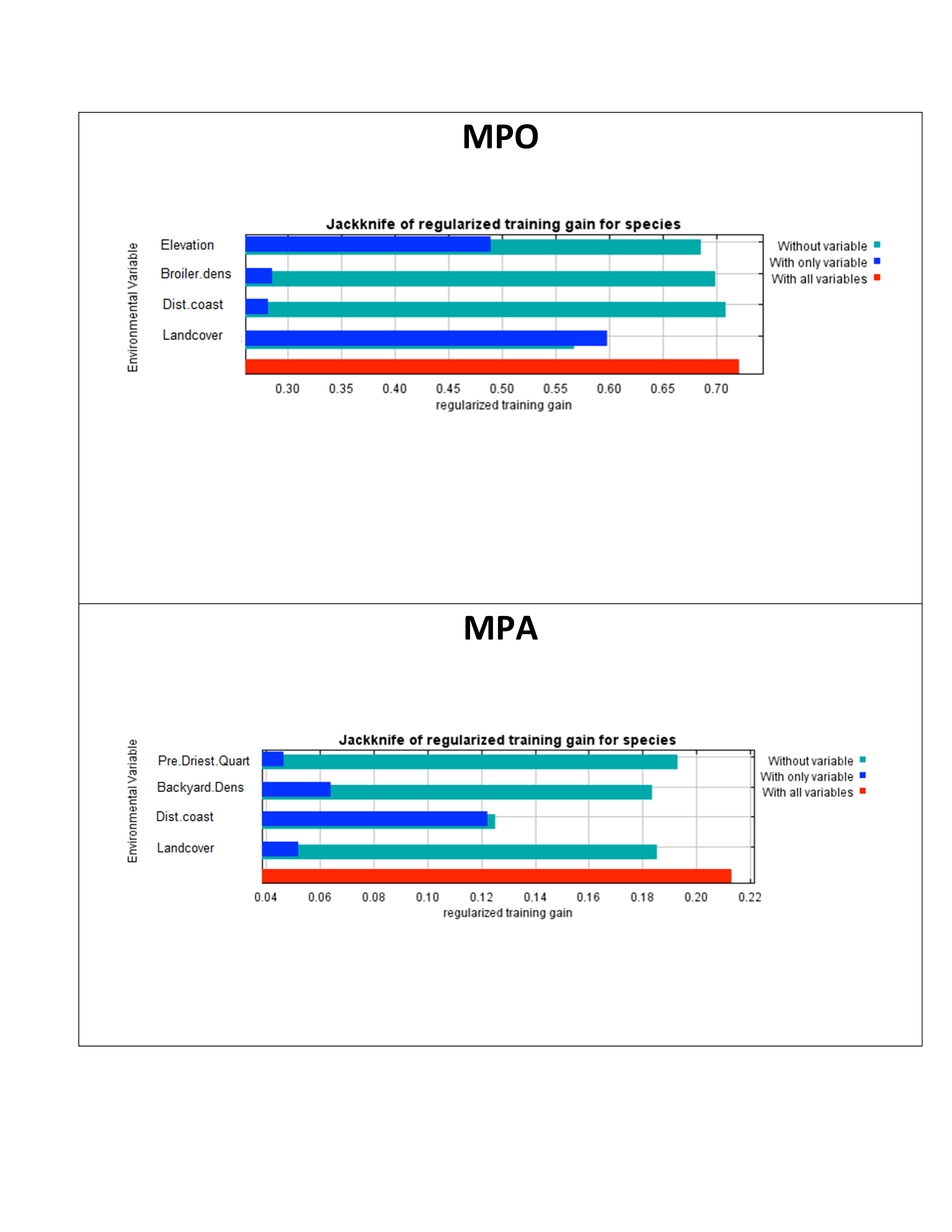

Supplement: S6 Fig — The red bar represents the overall training gain with all the included variables. The blue bar represents the training gain when using each variable in isolation. The clear blue bar is the training gain when the variable is excluded from the model. (TIF) [file pone.0190824.s009.tif]
